# Ultrasound Molecular Imaging of Renal Cell Carcinoma: VEGFR targeted therapy monitored with VEGFR1 and FSHR targeted microbubbles

**DOI:** 10.1038/s41598-020-64433-2

**Published:** 2020-04-30

**Authors:** Alexandre Ingels, Ingrid Leguerney, Paul-Henry Cournède, Jacques Irani, Sophie Ferlicot, Catherine Sébrié, Baya Benatsou, Laurène Jourdain, Stephanie Pitre-Champagnat, Jean-Jacques Patard, Nathalie Lassau

**Affiliations:** 1 IR4M, Univ. Paris-Sud, CNRS, Université Paris-Saclay, Orsay, France; 2 Department of Urology, CHU Henri Mondor, Créteil, France; 3 Gustave Roussy, University Paris-Saclay, Villejuif, France; 4 Lab MICS, Centrale Supélec, Université Paris-Saclay, Gif-sur-Yvette, France; 5 Department of Urology, CHU Kremlin-Bicêtre, Bicêtre, France; 6 Department of Pathology, Assistance Publique–Hôpitaux de Paris (AP-HP), Hôpitaux Universitaire Paris-Saclay, Hôpital de Bicêtre, France; 7 Service chirurgie urologique, Centre hospitalier, Mont-de-Marsan, France; 8 Imaging Department, Gustave Roussy, University Paris-Saclay, Villejuif, France

**Keywords:** Cancer models, Molecular imaging, Ultrasound

## Abstract

Recent treatment developments for metastatic renal cell carcinoma offer combinations of immunotherapies or immunotherapy associated with tyrosine kinase inhibitors (TKI). There is currently no argument to choose one solution or another. Easy-to-use markers to assess longitudinal responses to TKI are necessary to determine when to switch to immunotherapies. These new markers will enable an earlier adaptation of therapeutic strategy in order to prevent tumor development, unnecessary toxicity and financial costs. This study evaluates the potential of ultrasound molecular imaging to track the response to sunitinib in a clear cell renal carcinoma model (ccRCC). We used a patient-derived xenograft model for this imaging study. Mice harboring human ccRCC were randomized for sunitinib treatment vs. control. The tumors were imaged at days 0, 7, 14 and 28 with ultrasound molecular imaging. Signal enhancement was quantified and compared between the two groups after injections of non-targeted microbubbles and microbubbles targeting VEGFR1 and FSHR. The tumor growth of the sunitinib group was significantly slower. There was a significantly lower expression of both VEGFR-1 and FSHR molecular ultrasound imaging signals in the sunitinib group at all times of treatment (Days 7, 14 and 28). These results confirm the study hypothesis. There was no significant difference between the 2 groups for the non-targeted microbubble ultrasound signal. This study demonstrated for the first time the potential of VEGFR1 and FSHR, by ultrasound-based molecular imaging, to follow-up the longitudinal response to sunitinib in ccRCC. These results should trigger developments for clinical applications.

## Introduction

Renal Cell Carcinoma (RCC) represents 2–3% of all cancers. Incidence has increased over the last decades due to incidental detection by ultrasound (US) or computed tomography (CT) performed routinely for abdominal or back pains exploration^1^.

The clinical management of RCC presents two challenges due to epidemiology and therapeutics evolutions. On the one hand, the increased incidence of small tumors requires to differentiate benign from malignant tumors and to assess the aggressiveness of malignant tumors in order to avoid overtreatment of harmless lesions. On the other hand, the recent development of a vast arsenal of systemic treatment for metastatic stages requires the development of reliable, robust and easy-to-use biomarkers to evaluate the response to treatment of each new drug. This assessment would minimize side effects and unnecessary costs and prevent waste of time for non-responder patients.

Targeted systemic treatment against vascular endothelial growth factor (VEGFR), such as sunitinib, is the first line treatment recommended for treatment-naïve metastatic clear-cell RCC^2^.

There is currently no validated marker to predict the response to VEGFR targeted therapies in RCC. Assessment of treatment response is based on tumor volume variation according to the Response Evaluation Criteria In Solid Tumors (RECIST)^3^. Ultrasound is an easy–to-use, inexpensive, portable and fast imaging technique, without ionizing radiation and with real-time evaluation of the tumor. Contrast enhanced US imaging based on microbubbles detection has demonstrated its value for predicting sunitinib response in clinical trials^4,5^. There have been several studies evaluating ultrasound molecular imaging (USMI) in cancer to assess response to targeted therapies^6–15^. However, to our knowledge neither VEGFR-1 nor FSHR (Follicle Stimulating Hormone Receptor) have ever been explored so far.

The rationale for this study is that tumor molecular changes occur before vascularization changes and tumor shrinkage following VEGFR targeting therapies. Therefore, we investigated the differential expression of two molecular markers, VEGFR-1 and FSHR, between sunitinib and control groups of mice engrafted with patient-derived xenografts (PdX) of RCC during 4 weeks of treatment. We hypothesized that USMI signals and perfusion imaging parameters would be significantly lower in the treated group compared to the control group during the longitudinal follow-up US examinations (at weeks 1, 2 and 4 after initiation of sunitinib).

## Materials and Methods

All methods were performed in accordance with the relevant guidelines and regulations.

### Animals

Animal experiments were approved by the CEEA26 (ethics committee in animal experimentation) and the Ministry of Agriculture (approval number: APAFIS#8963‐2017022014433962) and performed under the conditions established by the European Community (Directive 2010/63/UE). The animals were raised and housed at the Institution Animal Care Facility. Balb/c Rag2–/–γc–/– female mice, highly immunodeficient, were chosen to promote greater tumor development. Three clear cell RCCs were collected from 3 nephrectomies of patients allowing the development of 3 experimental cohorts. In total, 33 mice were grafted and included in the protocol. The characteristics of the parental tumor and cohorts of animals are reported in Table 1.

**Table 1:** Tumor and cohort features. The number of mice per cohort and group is indicated with for each the tumor growth coefficient.

Cohort	Parental Tumor					Transplanted Cohort			
	Stage T	Stage N	Stage M	Führman Grade	Histology	Cohort size n (control/sunitinib)	Mean tumor growth coefficient Sunitinib	Mean tumor growth coefficient Control	p-value
1	1b	1	1	4	Clear cell	12 (6/6)	0.009	0.033	0.006
2	4	0	1	4	Clear cell Rhabdoid 50% Sarcomatoid 15%	14 (7/7)	0.024	0.037	0.183
3	3a	2	1	4	Clear cell Rhabdoid 50%	7 (3/4)	0.025	0.045	0.031

Patient Derived Xenograft models of RCC in mice: The method for grafting tissue sections and developing the RCC model has been accurately described in a previous study^16^. Briefly, an 8 mm core of fresh tumor was harvested in a non-cystic, non-necrotic area of a kidney tumor, immediately after surgical extirpation. The core was sliced into 300 µm homogeneous pieces with a dedicated device (Krumdieck tissue slicer, Alabama Research). Each slice was then implanted under the left kidney capsule of anesthetized mice under strict aseptic conditions. The engraftment was followed by weekly ultrasounds, and MRI to accurately measure the tumor size initially and after one month of treatment with sunitinib. Because the experiment involved patient’s derived tissue, the project was reviewed and accepted by Institut Gustave Roussy, Villejuif, France, Institutional Review Board. Every patient involved signed an informed consent.

**Figure 1:**
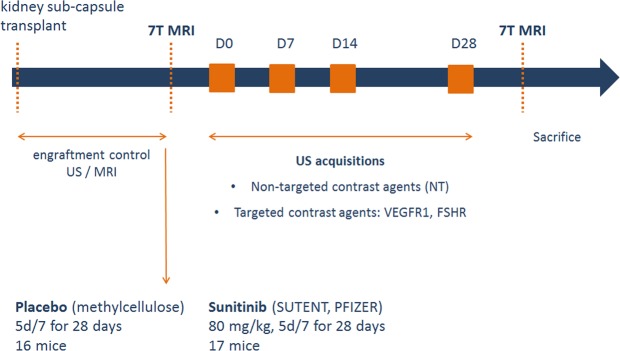
Study protocol. Once the transplant performed, mice were followed by imaging and then randomized into 2 groups: sunitinib or control. Imaging sessions included 7 T MRI imaging for accurate estimation of tumor volume, then ultrasound molecular imaging acquisitions at baseline and at 7, 14 and 28 days after initiation of treatment and a final 7TMRI for accurate estimation of the final volume. For each examination, non-targeted and targeted contrast agents for both markers (FSHR and VEGFR-1) were injected.

Sunitinib treatment and placebo groups (Fig. 1): The study started when tumors were measurable by ultrasound imaging and MRI. Each cohort was then divided into two homogeneous groups: one receiving a placebo solution (methocel 0.5%, vehicle solution) and the other receiving 80 mg/kg of sunitinib (Sutent, Pfizer, NYC, USA). The placebo and sunitinib were administered by gavage 5 days a week for 4 weeks.

**Figure 2:**
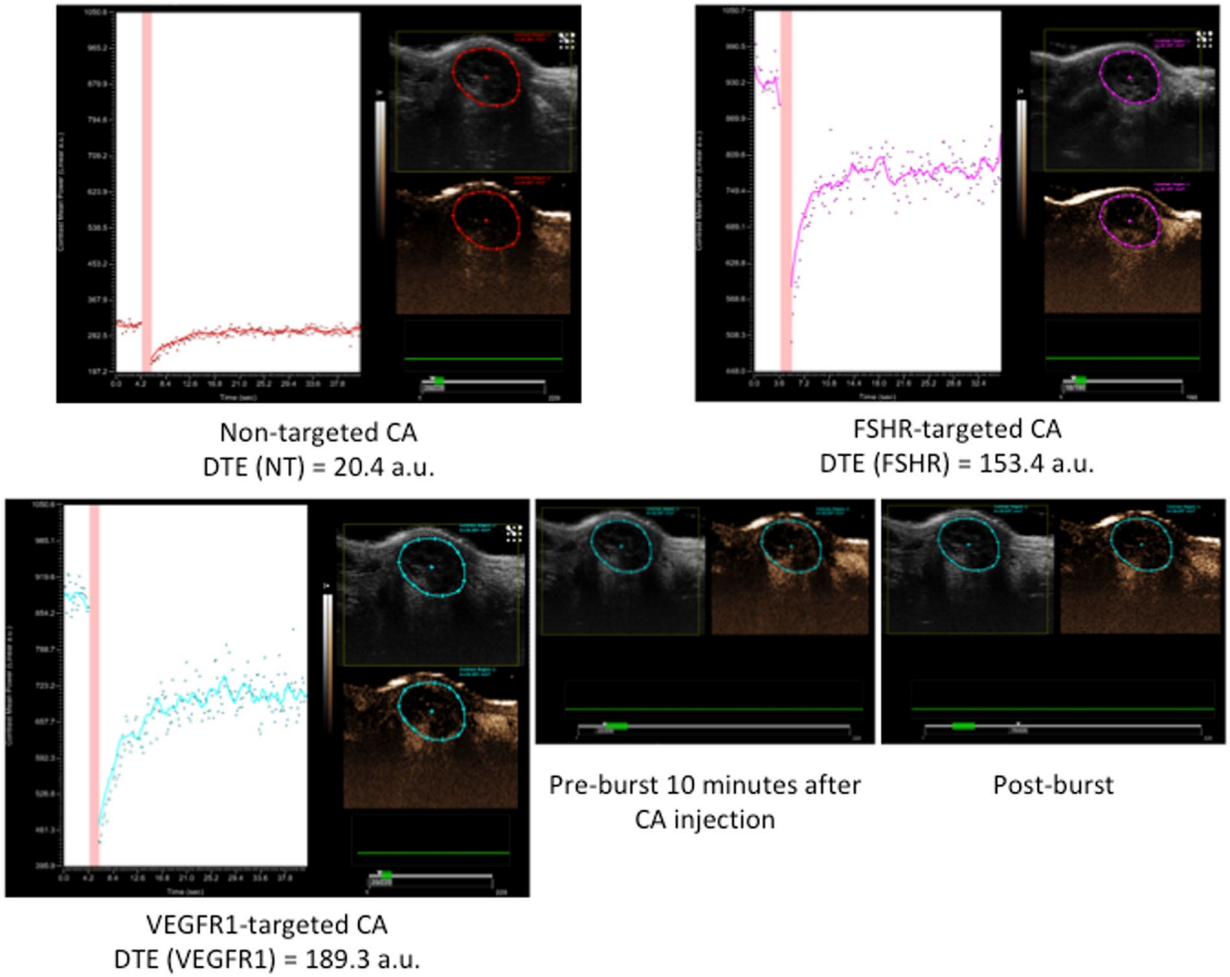
Examples of molecular imaging acquisitions on the same mouse. The ultrasound image is given for each contrast agent CA (non-targeted, targeted FSHR and VEGFR1), with the signal intensity measured within the tumor. The red band (green dash under the echo image) represents the destruction of the microbubbles (burst), with the signal measured before (10 minutes after injection of the CA) and the signal measured during replenishment. During the 10 minutes period following the injection of CA, the microbubbles attach to the receptors. Then, the microbubbles are destroyed before visualizing the reperfusion. The differential targeted enhancement DTE is calculated and corresponds to the difference between the signal at 10 minutes and the signal after replenishment. The quantity is proportional to the quantity of biomarkers in the region of interest.

US Molecular Imaging and biomarkers quantification (Fig. 2): The ultrasound imaging sessions were performed on a dedicated small animal US system (VEVO®2100, VisualSonics, Canada), using a 21 MHz central frequency linear probe (MS-250, VisualSonics, lateral and axial resolution of 165 and 75 µm, respectively). Before each imaging session, the mice were anesthetized by inhalation of isoflurane (2%) in room air (1.5 L/min). Their body temperature was monitored and kept constant using a heated platform during acquisitions (Bioseb, France). Tumor volumes were evaluated by 2D B-mode ultrasound imaging by measuring diameters in the transverse and longitudinal maximal planes as previously described in the literature^17,18^. The USMI and quantification of biomarker expression level were performed on the maximum 2D transverse section of each tumor using 50 µL of each contrast agents (CA): non-targeted (NT), VEGFR-1 and FSHR-targeted CA, injected in the retro-orbital vein. The CA are the MicroMarker microbubbles from VisualSonics (Toronto, Canada); each vial of targeted CA (target-ready CA) was prepared daily before injections. The MicroMarker target-ready vial was prepared by adding 700 µL of saline (0.9% sodium chloride). The solution was then stirred for ten seconds and then left at room temperature for 5 minutes. 20 µg of the biotinylated antibody (VEGFR1 or FSHR, Antibodies online) was diluted to a total volume of 300 µL with saline and injected into the vial of contrast agents. The 1 ml solution thus reconstituted was stirred for 1 minute and then left at room temperature for 15 minutes. For the non-targeted Micromarker, the vial was prepared by adding 700 µL of saline, then stirred for 1 minute and left for 10 minutes at room temperature^19^. A 30-minute interval between each CA injection allowed the microbubbles of the previous injection to be removed from the vascular system as described in a previous study^20^. The intensity of the CA signal was recorded as a function of time and the targeting was determined 10 minutes after the bolus injection to allow the targeted microbubbles to bind to their endothelial molecular receptors. The biomarker level was estimated using the VEVO®2100 software to quantify within the whole tumor area the Differential Targeted Enhancement (DTE) (value in arbitrary unit), which is an indicator of the number of microbubbles that adhere to molecular endothelial receptors. Briefly, the DTE was calculated by a destruction-replenishment method as the difference between the average intensities of the pre- (circulating and targeting CA) and post-(circulating CA) destruction signal in the same region of interest, as previously described in detail^20^. Tumor volumes and the targeted USMI were performed for the placebo and sunitinib groups at baseline (T0) and 7 (T7), 14 (T14) and 28 (T28) days after the start of the solution administrations.

**Figure 3:**
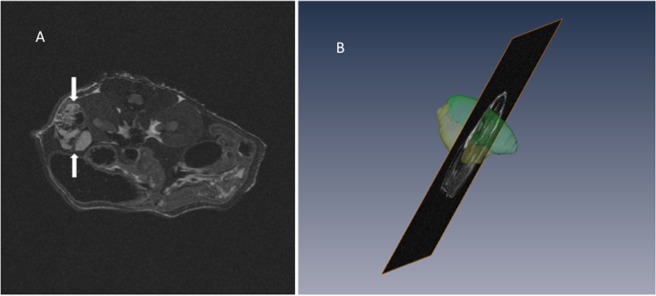
(**A**) Example of axial MRI T2W-image. The right and left kidneys are clearly visible with a contrasting tumor on the left kidney (between the arrows). The high signal intensity in the tumor reflects the presence of increased fluid in the tissues. (**B**) Example of 3D reconstruction of tumor (yellow) on the left kidney (green) using AMIRA software.

Magnetic resonance imaging (MRI): All the mice were imaged before and after the 28 days of treatment in order to perform anatomical images allowing a precise identification of the tumor in 3D with the left kidney, and thus to guide the choice of the section to be imaged by ultrasonic imaging. Mice were imaged under isoflurane anesthesia (induction 2%; flow rate 0.8% in 50% O_2_, 50% N_2_O) controlled on the basis of respiratory parameters. The body temperature was maintained at 37 °C using heated mattress. A 7-Tesla preclinical magnet (Bruker Avance Horizontal 7-T Bruker, Inc., Billerica, MA) equipped with a 35-mm-diameter “bird-cage” antenna was employed for MRI. The mouse body was positioned using scouting gradient echo images in the 3 orthogonal directions. After the shimming process, T2W-Axial Turbo-RARE (Rapid Acquisition with Relaxation Enhancement with T2-weighted) sequence was acquired [repetition time = 4800 ms; effective echo time =40 ms; echo spacing = 11 ms; rare factor = 9; 8 averages; not-contiguous 0.4 mm thick sections, with 0,1 mm slice Gap; 40 × 40 mm field of view; 384 × 384 matrix; pixel size = 104 μm^2^; sequence duration = 27 min]. Between 31 and 51 axial sections were acquired (Fig. 3A), depending on the size of the tumor and the left kidney. The total time spent by the mouse in the magnet was around 35 min. After each experiment, mice were released from anesthesia and returned to their home cages with free access to food and water. 3D volumes were reconstructed under the AMIRA software to measure tumor volume (Fig. 3B).

### Statistical analysis

The tumor growth model was based on the 4 tumor volume measurements (D0, D7, D14, D28). We used an exponential model V_i_(t) =V_i_°exp(a_i_t). Being a generalized linear model, we estimated the growth coefficient a_i_ and the origin value V_i_^0^ with a least square method. The model adjustment was estimated with the coefficient of determination R^2^ and the adjusted R^2^ values.

The mean value between two groups was compared with a Student t-test. A difference was considered significant when p < 0.05.

In the sunitinib group, we defined as non-responders the mice with tumor growth not statistically different from the average tumor growth of the control group. Being a posteriori analysis, the expressions of the markers were normalized on Day 0 so that the groups are comparable.

## Results

Cohorts and Tumor Growth: Regression analysis demonstrated a good adjustment between the model and tumor volume measurements (Mean R^2^ of 0.72, Mean adjusted R^2^ of 0.58). The model fits better to untreated mice (Mean R^2^ of 0.90); it is naturally less relevant when tumor growth is stabilized by the targeted treatment.

**Figure 4:**
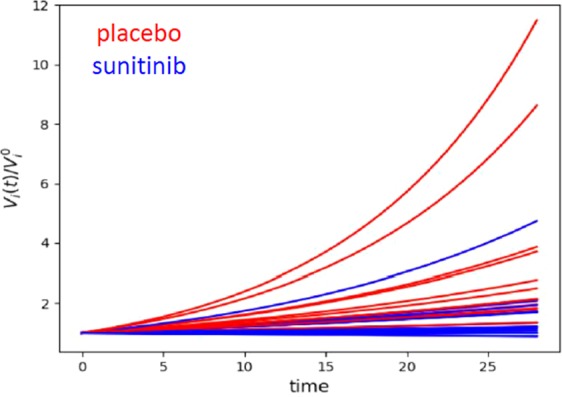
Tumor volume Vi(t) compared to the initial value Vi0 between days 0 to 28 of the tumor volume representing the exponential evolution of the tumor volume from which the tumor growth coefficient (ai) can be estimated from the 4 volume measurements (day 0, 7, 14 and 28), according to an exponential model V_i_(t)=V_i_^0^exp(a_i_t). Growth Rates of mice under placebo are represented in red and sunitinib in blue. We can clearly see the efficiency of sunitinib with slower growth rates.

Response to sunitinib: the sunitinib group had a significant slower tumor growth coefficient than the control group (0.012 vs 0.037; p = 0.0009) confirming the overall sunitinib efficacy (Fig. 4). However, when considering the RECIST criteria, we could not conclude in an objective response during the 28 days study period. Tumor growth in most sunitinib treated cases has stabilized or decreased compared to the control group. During the study period, the tumor size follow-up could not conclude in any treatment efficacy for the sunitinib. When comparing the individual tumor growth of sunitinib treated mice with the average control group, only 4 mice were considered as non-responders.

**Figure 5:**
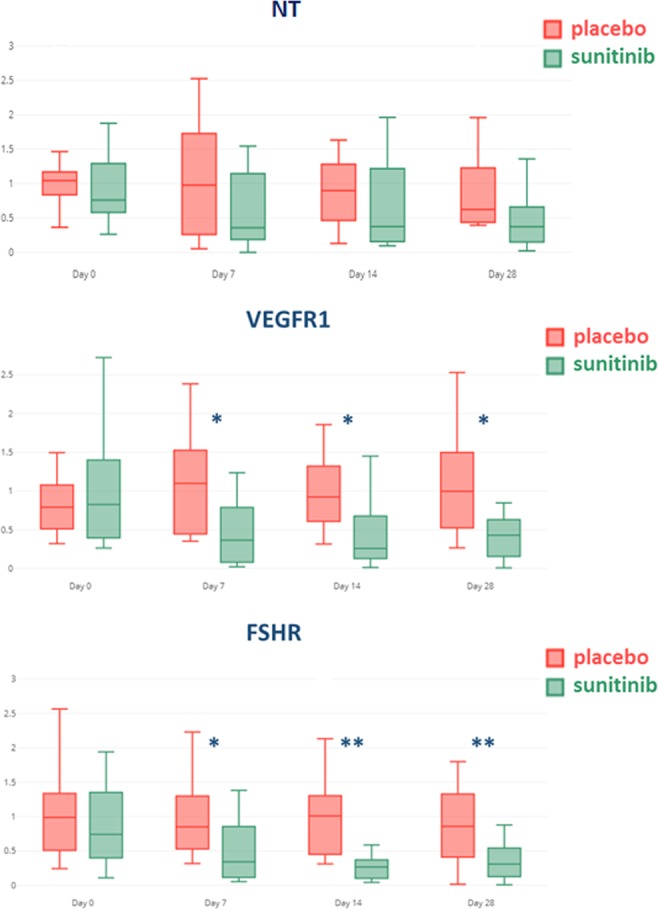
Distribution boxplot of the normalized expression level at each time point of Ultrasound Imaging Markers. The normalization was performed by dividing each measured value by the mean value of the whole population (treated and control) at T0, before treatment (up: Non Targeted microbubble, middle: VEGFR-1, down: FSHR). The boxplots compare placebo (red) and sunitinib (green) groups. We can see the differences are significant (*) at the 3 post-treatment initiation points (Day 7, 14 and 28) with the molecular imaging while non significant with the non targeted microbubble.

Markers differential expression: Differential markers expressions between the sunitinib and control group are reported in Table 2. There is a significant lower expression of both VEGFR-1 and FSHR molecular ultrasound imaging signals in the sunitinib group for all times of treatment (Day 7, 14 and 28). These results confirm the study hypothesis. There was no significant difference between the 2 groups for the non-targeted microbubble ultrasound signal (Fig. 5). We compared markers expression between the responders vs. the 4 non-responders inside the sunitinib group. We demonstrated a significant lower expression of VEGFR-1 (p-value Day 7 0.018, Day 14 0.032, Day 28 0.048) but no significant result for FSHR.

**Table 2:** p-values of the comparison between sunitinib and control groups for the 4 measurement points (days 0, 7, 14 and 28).

Variable	Non-Targeted (NT)	VEGFR-1	FSHR
p-value D0	0.301	0.98	0.98
p-value D7	0.164	0.021	0.046
p-value D14	0.181	0.038	0.001
p-value D28	0.094	0.028	0.010

## Discussion

The results of this study show that US molecular imaging using VEGFR-1 or FSHR-targeted microbubbles allows non-invasive assessment and longitudinal monitoring of sunitinib treatment. These imaging results were obtained on a PdX model of RCC.

Sunitinib is a Tyrosine Kinase Inhibitor (TKI), a VEGFR-targeted therapy validated for first line and second line treatment of metastatic clear cell RCC. Despite validated efficacy, some patients do not respond to VEGFR-targeted therapy or secondarily develop resistance. The median progression-free survival in the EFFECT trial was 9.9 months^2^. Therefore, access to a non-invasive marker for tracking initial and longitudinal response of patients undergoing this treatment is paramount. A molecular marker would present the interest of an earlier detection of non-responder patients than tumor volume measurements, the current gold standard. Indeed, the tumor shrinkage or expansion is often delayed in relation to the physiological phenomena and might not be completely reliable due to fibrosis, necrosis or cystic zones that constitute non-active tumor volumes. Such a marker would thus allow a quicker switch to an alternative treatment such as immune therapies and therefore to delay progression and avoid unnecessary side effects and costs related to TKI. Ultrasound imaging has many advantages compared to other techniques: widely available, easy to transport (bedside or office-based imaging), fast, relatively inexpensive and without ionizing radiation. For these reasons, we believe that ultrasound molecular imaging assessments might present the best clinical value.

A great advantage of molecular imaging compared with histological analysis from biopsies is the whole tumor molecular analysis. Indeed, a biopsy allows analysis from the restricted area where it has been gathered whereas USMI analyze the global marker expression of the entire tumor under the scope of the probe. This point is particularly relevant for RCC that are known for being very heterogeneous tumors^21^.

The PdX model of RCC is a real strength of this study compared to the usual model of xenograft from cell lines used for molecular imaging studies. Indeed, it has been shown that such a model allows reliable reproduction of the parental tumor over several generations. In contrast to cell line models, the tumor microenvironment and more specifically the peri-tumoral vascular system of the patient is implanted with the tumor cells. It has been shown that tumors implanted in mice harbor human vessels over several generations with this model^16^. In this way, this model particularly fits the goal of an endovascular tumor specific marker evaluation with USMI. However, there is the difficulty of greater variability between tumors.

Several studies reported pre-clinical validation of USMI to predict earlier TKI response in animal models of colorectal^6,8,10,14^, hepatocellular^7^, squamous cell^9^, pancreatic carcinoma^11^. In their study, Streeter *et al*. compared USMI signals in two groups of PDX mouse models: known responder tissue vs. known non-responder tissue for a targeted agent: MLN8237. The primary tumor was not detailed. The microbubbles targeted the αvβ3 integrin known to be associated with angiogenesis. They demonstrated that USMI signal was significantly different between the treated and the untreated group as early as day 2 in the responder cohort whereas the volume measurement was not different until day 14 at the end of follow-up^12^. Recently, two human clinical trials have evaluated USMI targeting VEGFR-2 in breast, ovarian^22^ and prostate cancers^23^. To our knowledge, only one prior study evaluated USMI for treatment response in renal cell carcinoma: Rojas *et al*. followed a cohort of ccRCC xenograft mice treated with either sunitinib or Notch pathway inhibitor GSI^15^. They demonstrated that USMI could track disease progression and assess functional changes in tumors before volume changes became apparent^15^.

Our study is the first to evaluate VEGFR-1 and FSHR markers by USMI. Surprisingly, whereas both VEGFR 1 and 2 are reported for playing key roles in angiogenesis and TKI targeting, only VEGFR-2 had been explored so far. The rationale for FSHR investigation is based on Radu *et al*. demonstrating that FSHR is selectively expressed on blood vessels surface of a wide range of malignant tumors including RCC^24^. Therefore this marker appears as a perfect candidate for USMI since ultrasonic microbubbles only circulate in blood vessels. Another study compared the expression of FSHR by immunofluorescence on nephrectomy samples of metastatic RCC patients subsequently treated with sunitinib. They demonstrated that the level of FSHR expression was predictive of the response to sunitinib treatment^25^. More recently, radioactive tracers conjugating a FSH probe with the ^18^F tracer has been developed and evaluated *in vivo* in a prostate PC3 cells mouse model. The authors demonstrated the method feasibility without longitudinal follow-up and treatment response assessment^26^.

Our results showed a significant difference between treated and untreated group for VEGFR-1 and FHSR expression using the non-invasive USMI method as early as the first post-treatment measurement (day 7). The difference between responders and non-responders was a secondary endpoint and was only found with the VEGFR-1 probe.

Some limitations have to be acknowledged. As a preclinical study, the size of the cohort was limited. Although we could demonstrate differential expression between treated and untreated groups for both VEGFR-1 and FSHR markers, we could only demonstrate differences between responders and non-responders with VEGFR-1. This last outcome has to be tempered due to a limited size of the non-responders group and ad-hoc analyzes exposing to selection bias. Our results demonstrate the FSHR and VEGFR-1 expression modification during the treatment (longitudinal analysis) and in comparison with the non-targeted agent. Unfortunately we failed to validate a correlation between molecular ultrasound imaging findings and post-sacrifice tumor’s marker expression analysis measured by immunohistochemistry. We have attended several protocols to get such a confirmation, including serial analysis of immunohistochemistry slides with slides marked with CD31 and VEGFR-1 or FSHR. We superposed the images in order to measure FSHR and VEGFR-1 expression in the vessel area that was expressing CD31. Unfortunately, these results were non-concluding.

This limitation is probably due to the difficulty of measuring the specific intra-luminal expression of the markers in the tumor vasculature by immunohistochemistry. The marker as we used it, is not directly translatable to clinical trials. We used the biotin-streptavidin complex to bind the antibody to the microbubble. We chose this solution because it is an easy way to explore new markers in a preclinical context. Such a complex presents anaphylactic risks and alternative solutions to anchor the antibody must be developed. This was already done for VEGFR-2 and the BR55 agent used in clinical trials^22,23^. The imaging was performed in a two-dimensional plane, requiring the selection of a tumor zone, not allowing to reflect the fluctuations of the expression of the marker in RCC, known as a very heterogeneous cancer^21^. This pitfall could be overcome with a whole tumor three-dimensional analyze.

In conclusion, our study suggests that molecular ultrasound imaging targeting FSHR or VEGFR-1 markers has real potential to follow-up patients with Renal Cell Carcinoma undergoing tyrosine kinase inhibitor treatment such as sunitinib. This easy-to-use, non-invasive technique would be of great interest in clinical practice to avoid unnecessary treatment and complications and to switch earlier to other drugs. These results should trigger further research to explore VEGFR-1 and FSHR targeting potential in MI for other cancers and in clinical settings for kidney cancer.

## References

[1] Patard J-J, Rodriguez A, Rioux-Leclercq N, Guillé F, Lobel B (2002). Prognostic significance of the mode of detection in renal tumours. BJU Int..

[2] Ljungberg B (2015). EAU guidelines on renal cell carcinoma: 2014 update. Eur. Urol..

[3] Nishino M, Jagannathan JP, Ramaiya NH, Van den Abbeele AD (2010). Revised RECIST guideline version 1.1: What oncologists want to know and what radiologists need to know. AJR Am. J. Roentgenol..

[4] Lassau N (2010). Metastatic renal cell carcinoma treated with sunitinib: early evaluation of treatment response using dynamic contrast-enhanced ultrasonography. Clin. Cancer Res. Off. J. Am. Assoc. Cancer Res..

[5] Lassau N (2014). Validation of dynamic contrast-enhanced ultrasound in predicting outcomes of antiangiogenic therapy for solid tumors: the French multicenter support for innovative and expensive techniques study. Invest. Radiol..

[6] Wang H, Lutz AM, Hristov D, Tian L, Willmann JK (2017). Intra-Animal Comparison between Three-dimensional Molecularly Targeted US and Three-dimensional Dynamic Contrast-enhanced US for Early Antiangiogenic Treatment Assessment in Colon Cancer. Radiology.

[7] Baron Toaldo M (2015). Use of VEGFR-2 targeted ultrasound contrast agent for the early evaluation of response to sorafenib in a mouse model of hepatocellular carcinoma. Mol. Imaging Biol. MIB Off. Publ. Acad. Mol. Imaging.

[8] Eschbach RS (2017). Contrast-Enhanced Ultrasound with VEGFR2-Targeted Microbubbles for Monitoring Regorafenib Therapy Effects in Experimental Colorectal Adenocarcinomas in Rats with DCE-MRI and Immunohistochemical Validation. PloS One.

[9] Baetke SC (2016). Squamous Cell Carcinoma Xenografts: Use of VEGFR2-targeted Microbubbles for Combined Functional and Molecular US to Monitor Antiangiogenic Therapy Effects. Radiology.

[10] Wang H, Kaneko OF, Tian L, Hristov D, Willmann JK (2015). Three-dimensional ultrasound molecular imaging of angiogenesis in colon cancer using a clinical matrix array ultrasound transducer. Invest. Radiol..

[11] Pysz MA (2015). Vascular endothelial growth factor receptor type 2-targeted contrast-enhanced US of pancreatic cancer neovasculature in a genetically engineered mouse model: potential for earlier detection. Radiology.

[12] Streeter JE, Herrera-Loeza SG, Neel NF, Yeh JJ, Dayton PA (2013). A comparative evaluation of ultrasound molecular imaging, perfusion imaging, and volume measurements in evaluating response to therapy in patient-derived xenografts. Technol. Cancer Res. Treat..

[13] Sirsi SR (2012). Contrast ultrasound imaging for identification of early responder tumor models to anti-angiogenic therapy. Ultrasound Med. Biol..

[14] Zhou J (2016). VEGFR2-Targeted Three-Dimensional Ultrasound Imaging Can Predict Responses to Antiangiogenic Therapy in Preclinical Models of Colon Cancer. Cancer Res..

[15] Rojas JD (2018). Ultrasound Molecular Imaging of VEGFR-2 in Clear-Cell Renal Cell Carcinoma Tracks Disease Response to Antiangiogenic and Notch-Inhibition Therapy. Theranostics.

[16] Thong AE (2014). Tissue slice grafts of human renal cell carcinoma: an authentic preclinical model with high engraftment rate and metastatic potential. Urol. Oncol..

[17] Leguerney I (2012). Combining functional imaging and interstitial pressure measurements to evaluate two anti-angiogenic treatments. Invest. New Drugs.

[18] Lavisse S (2008). Early quantitative evaluation of a tumor vasculature disruptive agent AVE8062 using dynamic contrast-enhanced ultrasonography. Invest. Radiol..

[19] Willmann JK (2008). US imaging of tumor angiogenesis with microbubbles targeted to vascular endothelial growth factor receptor type 2 in mice. Radiology.

[20] Leguerney I (2015). Molecular ultrasound imaging using contrast agents targeting endoglin, vascular endothelial growth factor receptor 2 and integrin. Ultrasound Med. Biol..

[21] Gerlinger M (2012). Intratumor heterogeneity and branched evolution revealed by multiregion sequencing. N. Engl. J. Med..

[22] Willmann JK (2017). Ultrasound Molecular Imaging With BR55 in Patients With Breast and Ovarian Lesions: First-in-Human Results. J. Clin. Oncol. Off. J. Am. Soc. Clin. Oncol..

[23] Smeenge M (2017). First-in-Human Ultrasound Molecular Imaging With a VEGFR2-Specific Ultrasound Molecular Contrast Agent (BR55) in Prostate Cancer: A Safety and Feasibility Pilot Study. Invest. Radiol..

[24] Radu A (2010). Expression of follicle-stimulating hormone receptor in tumor blood vessels. N. Engl. J. Med..

[25] Siraj MA, Pichon C, Radu A, Ghinea N (2012). Endothelial follicle stimulating hormone receptor in primary kidney cancer correlates with subsequent response to sunitinib. J. Cell. Mol. Med..

[26] Xu Y (2014). Pilot study of a novel (18)F-labeled FSHR probe for tumor imaging. Mol. Imaging Biol. MIB Off. Publ. Acad. Mol. Imaging.

